# Enhancing long COVID care in general practice: A qualitative study

**DOI:** 10.1371/journal.pone.0306077

**Published:** 2024-06-26

**Authors:** John Broughan, Emīls Sietiņš, Ka Yuet Emily Siu, Nia Clendennen, Claire Collins, Ronan Fawsitt, John S. Lambert, Stefano Savinelli, Stephanie Skeffington, Geoff McCombe, Walter Cullen

**Affiliations:** 1 Clinical Research Centre, School of Medicine, University College Dublin, Dublin, Ireland; 2 School of Medicine, University College Dublin, Dublin, Ireland; 3 The Coombe Family Practice, Dublin, Ireland; 4 Irish College of General Practitioners, Dublin, Ireland; 5 Department of Public Health and Primary Care, Faculty of Medicine and Health Sciences, Ghent University, Gent, Belgium; 6 Castle Gardens Surgery, Kilkenny, Ireland; 7 Ireland East Hospital Group, Dublin, Ireland; 8 Mater Misericordiae University Hospital Dublin, Dublin, Ireland; 9 The Rotunda Hospital, Dublin, Ireland; 10 St Vincent’s University Hospital, Dublin, Ireland; Vrije Universiteit Brussel, BELGIUM

## Abstract

**Introduction:**

Research suggests that general practice can play an important role in managing long COVID. However, studies investigating the perspectives of general practitioners (GPs) and patients are lacking and knowledge regarding optimal long COVID care in general practice is therefore limited.

**Aim:**

To investigate GPs’ and patients’ perspectives on the topic of long COVID and its management in general practice.

**Methods:**

Brief questionnaires (GP n = 11, Patient n = 7) and in-depth semi-structured interviews (GP n = 10, Patient n = 7) were conducted with GPs and patients from Irish general practices during July 2022-January 2023. Interviews were conducted via telephone and audio recordings were transcribed. A phenomenological analysis involving reflexive thematic analysis and constant comparison techniques was adopted.

**Results:**

Analysis of interviews with GPs (male = 7, female = 3; median age = 50yrs (IQR = 39.5–56)) and patients (males = 2, female = 5; median age = 58yrs (IQR = 45-62yrs) generated four themes. These were (1) Complex presentations (2) the value of standardising care, (3) choosing the right path, and (4) supportive and collaborative doctor-patient relationships. Strong agreement was observed among GPs and patients regarding the need for holistic and integrated multidisciplinary care. Supportive and collaborative doctor-patient relationships were largely well received by GPs and patients also. GPs strongly endorsed standardising long COVID care operations.

**Conclusion:**

GPs and patients indicated that structured, integrated, and collaborative care can help optimise long COVID management in general practice. GPs are advised to incorporate these elements into their long COVID care practices going forward. Future research examining stakeholder’s perspectives using larger and longitudinal samples is advised to enhance the generalisability of evidence in this area.

## Introduction

Long COVID, often referred to as post-COVID conditions, post-COVID-19 syndrome, post-Acute COVID-19, Long Haul COVID and several other similar terms, is a long-term condition that can arise following COVID-19 infection. Long COVID diagnoses are typically given when patients continue to experience symptoms resulting from the COVID-19 virus three months following infection. Symptoms commonly include chronic fatigue, cognitive problems (e.g., difficulty concentrating), and shortness of breath. However, the nature of long COVID symptoms, their underpinning biological mechanisms, distribution within populations, symptom severity, and symptom duration is highly varied between affected individuals [1–3]. For instance, research has shown that the chances of developing long COVID symptoms are lower when affected individuals are infected by the Omicron COVID-19 variant [4], that there is no universal pathogenic biological mechanism for long COVID, and that women, ethnic, sexual, and gender minorities, as well as people without college education, are more likely to experience long COVID symptoms and related disability.

An increasing amount of research has been published championing the role of general practitioners (GPs) in managing patients with long COVID. Patients with long COVID can have complex and ongoing care needs. That is, these patients often experience long-term morbidities and co / multi-morbodities including respirtory, cardiological, neurological, psychological, gestational and social functioning problems. Studies have reported that GPs can help address these kinds of problems by capitalizing on their (i.e., GPs) unique position as the initial, most holistic, and most longitudinal point of contact for many patients requiring care [1, 2, 5–8]. Research also indicates that GPs can enhance long COVID care by maximizing the potential of preventative, diagnostic, and treatment solutions available to them (e.g., blood tests, diagnostic imaging technology, comprehensive patient records in Electronic Health Records), whether directly available or through referral [9–19]. Clear and compassionate communication between GPs and patients has also been stressed as key to ensuring optimal long COVID management [7, 16, 20–23]. Other notable phenomena reported to enhance long COVID care include GPs who proactively help patients navigate care pathways [7, 10, 24], the unique holistic contributions that GPs can bring to multidisciplinary care teams [2, 25–29], and the value of GPs who keep up to date with developments in published long COVID research and care guidelines [16, 18, 25, 30, 31].

Qualitative research has an important role in determining the feasibility and acceptability of healthcare, and long COVID is no different in this respect [32]. Of the eight qualitative studies found relating to long COVID in general practice and primary care, two simultaneously examined the perspectives of primary care practitioners (not GPs) and patients [28, 33]. Other studies examined the views of GPs [34], general practice patients [10], patients recruited from the community rather than from primary care or general practice [30], patients referred to a primary care integrated psychology service [24], doctors from social media (of which eight were GPs) [35], and members of the general public recruited via social media [20]. The largest study sample containing both general practice / primary care practitioner and patient participants involved 198 paricipants (patient n = 126; primary care practitioner n = 72) and employed content analysis of open-ended survey responses rather than in-depth interviews [28]. The other study containing clinician and patient participants recruited a sample size of 52 which included 40 patients and 12 healthcare providers, albeit only two of these providers were GPs [33]. Four studies were conducted in the UK [20, 24, 33, 35], while single studies were conducted in Germany [28], Belgium [10], Slovenia [34], and the USA [30]. In sum, the long COVID qualitative research field has made progress. However, its limitations regarding sampling and chosen analysis approaches mean that there remains a need for in-depth interview-based qualitative studies that simultaneously elucidate the perspectives of GPs and patients in healthcare delivery contexts.

The findings of the qualitative studies conducted to date regularly point to the importance of ‘long COVID aware’ clinicians and stress the worth of providers that (1) inform person centred care and recovery by listening closely to patients with long COVID [20, 30, 33, 35], (2) overcome psychological difficulties caused by uncertainty regarding long COVID’s often unpredictable symptom progression, treatment course, and functional impacts [20, 24, 28, 34, 35], and (3) facilitate high standards of longitudinal, multidisciplinary, and integrated patient care [10, 20, 28, 30, 33, 35]. Thus, the solutions proposed by these qualitative studies are largely like those reported in the wider research around primary care management of long COVID. The qualitative findings are unique meanwhile in the sense that they offer deeper insights into the psychological and interpersonal factors underpinning the phenomenon of long COVID in primary / community care contexts. For example, in their 2022 paper ‘Opportunities to Improve Long COVID Care: Implications from Semi-structured Interviews with Black Patients’, Bergmans et al highlighted the negative mental health consequences of family physicians dismissing their patients’ beliefs that they may have long COVID [30]. A notable limitation of the available qualitative research in the area however is the paucity of studies focusing on long COVID in general practice settings, particularly studies comparing GP and patient perspectives on the issue.

### The present study

Much is still unknown regarding stakeholder perspectives on long COVID care in primary care settings. An insufficient number of qualitative studies have assessed long COVID care, fewer still in general practice, and no studies have simultaneously compared the perspectives of GPs and GP patients. Important issues such optimal approaches to care standards in general practice, the addressing of inequities among patient sub-populations, and long COVID referall pathway experiences require examination from both doctor and patient perspecitves if they are to be fully comprehended and actioned upon. Thus, there is a need for continued qualitative research to inform future research, practice, and policy in this area. To that end, this study involved conducting brief questionnaires and in-depth semi-structured qualitative interviews with GPs and general practice patients in Ireland. The study focused on answering the questions, “What has been the experience of long COVID in general practice?” and “How can general practice enhance long COVID care and care outcomes?”

## Methods

### Setting, participants, and sampling

This study was conducted with practitioners and patients recruited from the Ireland East Hospital Group / UCD School of Medicine’s General Practice Research Network (‘The Network’). The Network consists of 15 research active general practices focusing on wide ranging issues affecting general practice. Purposeful sampling that aimed to recruit as many participants as were required to achieve data saturation was used to recruit study participants, namely GPs from the Network and patients with long COVID attending their practices. All Network member practices were sent study invitations including comprehensive study information and an informed consent form to sign and return to the reseach team via post or email. Practices that agreed to participate were subsequently asked to purposefully identify and invite patients with long COVID attending their practice to take part in the study. Specifically, practices were asked to invite adult patients (18yrs+) that had been identified as either (a) having received a positive COVID-19 PCR / Antigen test or (b) having been clinically diagnosed with COVID-19. GPs were also asked to ensure that particularly vulnerable patients (e.g., people with profound disability, older persons, socially marginalised groups) would only be invited to take part if they had capacity to provide informed consent. If these criteria were met, GPs were asked to provide patients with contact details for the research team, who at this point provided patients via post and /or email with full study information (e.g., study information sheet), obtained their informed consent, and scheduled their involvement in the study. GPs received a fee for participating in the study. Patients were not given any material incentive for taking part.

### Data collection and management

Brief questionnaires, one for GPs and one for patients, were sent to participants via post and/or email from May 2022 to January 2023. Participants were asked to return completed brief questionnaires to the research team, either via prepaid postal envelope or email. Questionnaire data was extracted by the research team from completed paper and electronic response forms which were returned by participants between June 2022 and January 2023. The data was then entered in pseudonymised format into Microsoft Excel and IBM SPSS Statistics 27 for data cleaning and descriptive statistical analysis. Semi-structured interviews with the same participants were conducted over telephone by JB and KYES from 03/06/2022 to 27/01/2023. Interviews were audio recorded and transcribed. Transcripts were pseudonymised prior to analysis to ensure confidentiality. All electronic data was stored on encrypted files (Microsoft Excel, Word, NVivo 12 and SPSS 27) and computers belonging to research team members. Paper data (e.g., posted consent forms and questionnaire responses) were stored in locked filing cabinets in locked rooms at the host University.

### Study instruments

#### Brief questionnaires

Brief bespoke questionnaires informed by the findings of previous research in the areas of long COVID and GP long COVID management [e.g., 1, 2, 25] were used to establish study participants’ demographic profiles and introductory context regarding their long COVID views and experiences. The GP questionnaire included nine items querying GPs’ age, gender, practice location, the numbers of patients attending their practice, the type and number of staff working at their practice, their satisfaction with care at the practice, and Long COVID care initiatives they would like to see implemented (see S1 Appendix). Patient questionnaires contained eleven items. These items included open and closed response questions and asked about participants’ age, gender, long COVID symptoms and their health impacts, experiences with long COVID care in general practice, and potential long COVID care solutions in general practice (see S2 Appendix).

#### Semi-structured interviews

Semi-structured interview schedules were used for GPs and patients (see S3 and S4 Appendices). Interview questions were prompted by participants’ prior responses to the brief questionnaires, thus facilitating capacity for triangulation between the questionairre and interview findings.

### Qualitative approach and research paradigm

A phenomenological approach grounded in Heideggerian phenomenological theory [36] was used to provide a theoretical framework guiding analysis of the qualitative interview data. Heideggarian phenomenological theory highlights the subjectivity of individuals (including analysts) as they exist in the world (Dasein), the role that their subjectivity plays in the creation of meaning or knowledge, and the importance of of reflexivity among analysts to ensure that they are conscious of and transparent about the influence of their own biases during the interpretation process. In line with approach, emphasis was placed on reflexively generating themes that illustrated participants’ views and experiences regarding long COVID and its management in general practice. Phenomenological assumptions (e.g., the role of subjectivity in meaning creation, the value of reflexive analysis) often guide service-based investigations like the one conducted in this study as they can provide valuable insights regarding key stakeholders’ service-related perspectives [37–42]. The Heideggerian method was further underpinned by a grounded theory influenced constant comparison analytical strategy which was employed. This strategy spanned each of the six phases the entire data analysis process, and focused on establishing greater context around the study problem by examining similarities and differences within and between the perspectives of participants (e.g., between GPs and patients) where applicable, as well as similarities and differences within and between the interpretations of researchers when analysing the data [43]. Braun and Clarke’s six-phased Reflexive Thematic Analysis (RTA) approach was also incorporated to provide a systematic and accessible methodological framework for the analysis. In addition, RTA is theoretically compatible with phenomenological worldviews, particularly Heidegger’s phenomenology as it also endorses the value of reflexivity on the analysts’ part, and the constant comparison method [43, 44]. The ‘Standards for reporting qualitative research: a synthesis of recommendations’ (SRQR) guidelines were also consulted for added methodological rigour where relevant [45]. Interview data with GPs and GP patients combined was analysed by three researchers (JB, EKYS, ES).

### Researcher characteristics and reflexivity

The first author (JB) is a General Practice Research Coordinator at the Clinical Research Centre in the School of Medicine, University College Dublin (UCD), Ireland. He is also a PhD student at the UCD School of Medicine and his prior academic background is in Psychology. This study was conducted as part of his PhD by research programme. His PhD research aims to advance knowledge regarding how long COVID care can be enhanced in general practice. The study team also contains fellow students and research staff at the UCD School of Medicine, and healthcare professionals working at academic and healthcare service bodies in Ireland The study participants were GPs and patients in the General Practice Research Network mentioned earlier. The ‘Network’ member practices were recruited from the professional networks of the research team, particularly principal investigators WC and RF. To the best of our knowledge, patients taking part in the study had no prior relationship with members of the research team.

### Ethics

The study was approved by the Irish College of General Practitioner’s Research Ethics Committee on 13/01/2022 (ICGP_REC_21_0060).

## Results

Fifteen GPs in the GP Research Network were invited to take part in the study and eleven did so. Seven patients from four of the eleven participating practices took part. It is unclear how many patients participating GPs invited to take part in the study.

### Brief questionnaire findings

#### GPs

Eight males and three females completed the study’s brief questionnaires. Their median age was 52.5yrs (IQR = 41.3–57.8). Four GPs described their practice as ‘urban’, one GP described their practice as ‘rural’, and five said their practice was ‘mixed urban / rural’. The median number of GPs working at their practices was five (IQR = 4–7). Other staff working at the practices included practice nurses (n = 11), one psychologist / counsellor, and two physiotherapists. The median approximate number of patients attending the practices was 6, 467 (IQR = 4,625–10,875), and the reported median approximate number of patients that had COVID-19 attending the practices was 338.5 (IQR = 204.8–956.8)

#### Patients

Of the seven patients that completed brief questionnaires, two were male and five were female. Their median age was 58 years (IQR = 45–62). One patient said they had been experiencing long COVID symptoms for two to four weeks, one reported they had symptoms for 12 weeks to six months, and five said they had symptoms for more than a year.

**Fig 1:**
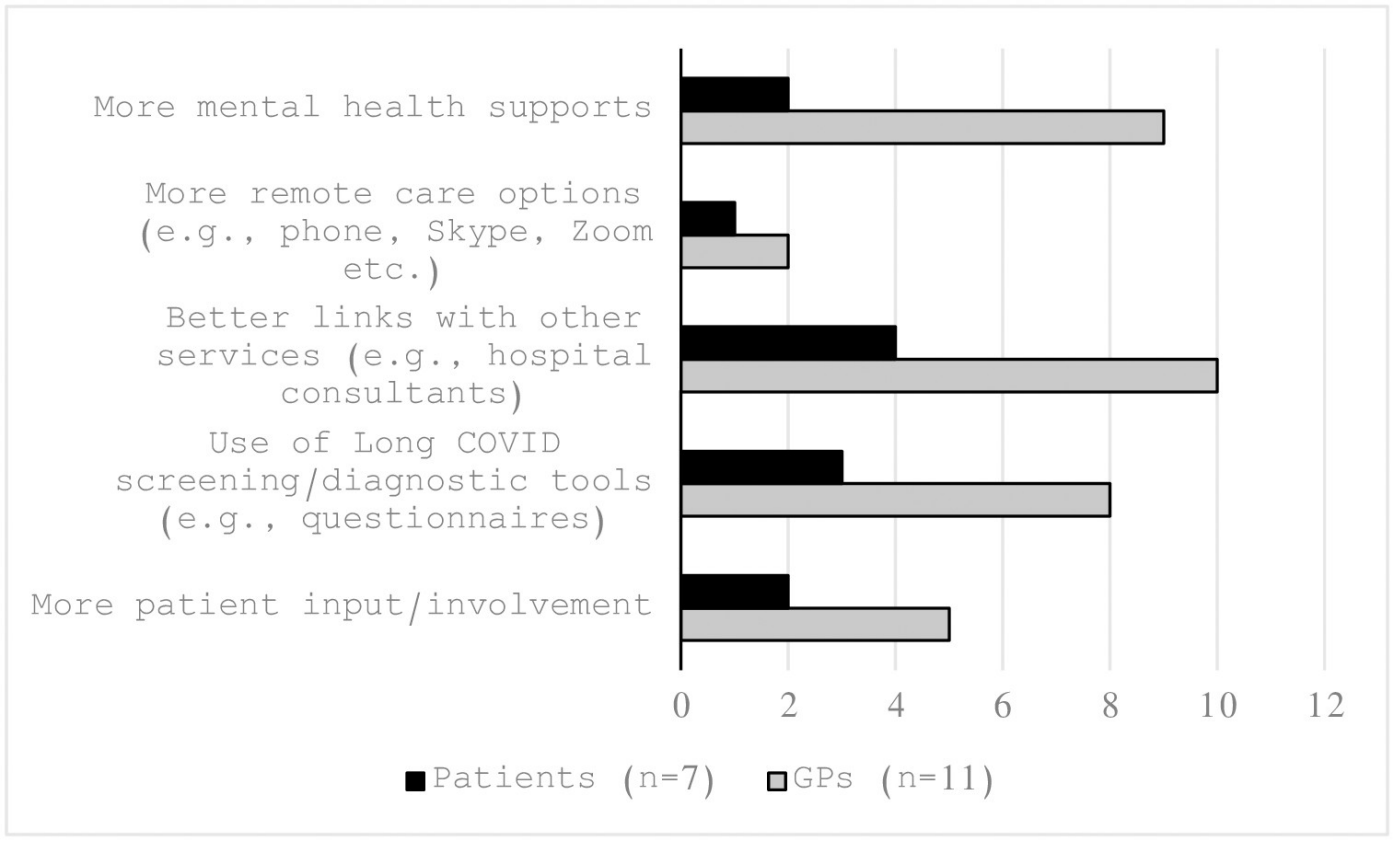
Interventions I would like to see implemented in GP long COVID care.

Table 1 outlines GP and patient responses to brief questionnaire Likert scale questions. Fig 1 shows GP long COVID care interventions that GPs and patients would like to see implemented.

**Table 1:** Frequency of GP and patient responses to brief questionnaire Likert scale items.

**GPs**					
Item	Strongly disagree	Disagree	Neither agree nor disagree	Agree	Strongly agree
I am satisfied with the COVID-19 care provided at my practice	1	1	4	4	1
**Patients**					
Acute COVID-19 (i.e., first 2–3 weeks following COVID-19 infection) made me feel unwell	0	0	0	2	5
Long COVID made me feel unwell	0	0	0	3	4
Long COVID negatively impacted my physical health	0	0	0	1	5
Long COVID negatively impacted my mental health	0	1	2	0	4
Long COVID negatively impacted my quality of life	1	0	0	3	3
I am satisfied with the Long COVID care I received from my GP	0	0	2	1	4

### Qualitative interview findings

In-depth semi-structured qualitative interviews were conducted with 10 of the 11 participating GPs and all seven participating patients. Four themes were generated applying Braun and Clarke’s Reflexive Thematic Analysis analytical approach. These were (1) complex presentations, (2) the value of standardising care, (3) choosing the right path, and (4) supportive and collaborative doctor-patient relationships. Theme 1 had two sub-themes (“Symptoms are often confounded by comorbidities” & “Long COVID’s psychosocial impacts must also be addressed”), while Theme 4 had one sub-theme (“Empowering patients”).

#### Theme 1—Complex presentations

Interviews with both GPs and patients both indicated that long COVID symptoms are typically numerous, diverse, and highly varied between individuals.

“*I think tiredness*, *cardiovascular symptoms*, *and neurological symptoms (are common)*. *In the short term*, *lingering respiratory symptoms*, *so persistent cough or breathlessness*. *And in the medium term*, *it seems to be cardiovascular*, *neurological*, *and general malaise type problems*.”

–*GP 4*

Descriptions of symptom severity and duration among patients with long COVID varied considerably.

“…*it is very variable in terms of how long it lasts*. *You might see it for a few months or (patients) might be coming in saying that they’ve been out for almost a year*. *But the long-lasting ones would certainly be in the minority*. *And we haven’t found an onerous burden in terms of patients coming in with long COVID symptoms*, *but I think for the few who do have them it’s been very distressing*.”

–*GP 7*

“*I realised that I had a lot of other issues*, *but that I only had one primary issue at any time*. *So*, *when the tremors and my brain*, *my neurology got really bad*, *my respiratory came second*. *So*, *as the chronic cough became manageable*, *the other one showed its ugly head*.”

–*Patient 4*

*Sub-theme i—Symptoms are often confounded by comorbidities*. Co-morbidities often complicated things further. Uncertainty and disagreement around whether to attribute symptoms to long COVID or pre-existing health conditions were common among participants.

“*I’ve probably met three or four (patients) who think they have long COVID*, *and I haven’t really been able to convince myself that they have it and our approach in those situations is*, *first*, *so it’s always a diagnosis of exclusion*, *so you find yourself doing tests*.”

–*GP 14*

Some patients said that long COVID exacerbated their pre-existing health problems. Diagnostics occasionally confirmed previously undetected health issues among patients seeking care with long COVID complaints.

“*My impression of long COVID is that if you have something small that’s niggling*, *it seems to be speeding it up*.”

–*Patient 5*

*Sub-theme ii—Long COVID’s psychosocial impacts must also be addressed*. Psychosocial issues stemming from long COVID’s adverse impacts on daily functioning and problematic social perceptions of long COVID were also mentioned by GPs and patients. These issues included reduced quality of life, anxiety, depression, and suicidal ideation.

“…*I’m thinking of a couple of patients who struggled to get back to work and were struggling mentally with feeling sick for so long… Some people bounce back very quickly*, *and others take longer*… *And a big part of the support that they require is psychological support*.”

–*GP 9*

“*The tiredness then leading to me being so cranky*, *being so irritated about things…that takes away from your life so much*. *That was the big thing*.”

–*Patient 1*

*“*…*my mental health is getting to me*. *I’m getting very down in myself because I can’t do what I was doing (before)*.”

–*Patient 3*

Having their symptoms misunderstood or not taken seriously by family, friends, and health professionals were frequent sources of distress for interviewed patients, with feelings of alienation common among those affected.

“…*don’t leave us feeling that you don’t care… that’s probably the worst feeling in the world*, *when you walk away because then you have the other side of it*, *‘Is this in my head*, *does he think I’m making this up*?’”

–*Patient 5*

Overall, the complexity of long COVID symptoms and psychosocial consequences among patients represented a major challenge for GPs searching for clinical solutions and patients seeking care.

#### Theme 2—The value of standardising care

Interviewed GPs often indicated that long COVID could be better addressed in general practice by mainstreaming standardised long COVID definitions.

“*Well*, *firstly*, *it’s actually quite difficult to diagnose long COVID in general practice*, *we don’t really have a definition that we work to*.”

–*GP 4*

Standardising care procedures was also recommended.

“*…if you could objectify the patient experience (using a standardised long COVID assessment tool)…it will allow you to be scientific about it and say*, *‘Well*, *you are better than this patient and worse that that patient*, *you’re better than you were a month ago*, *you’re worse than you were a month ago…’…I imagine a tool like that*, *apart from making the patient feel validated*, *it probably enhances the consultation process because the doctor doesn’t feel blindsided or impotent in the face of a challenging situation*. *On the other hand*, *the tools can be misleading…but used judiciously*, *they can be an adjunct to managing the patient*…”

–*GP 14*

As was the standardisation of integrated multidisciplinary pathways.

“*So*, *I feel in some respects there is no true pathway to follow for someone with long COVID… you’re nearly swimming in the sea without any support or any compass at all*.”

–*GP 10*

Patients had relatively little to say with regards to standardised definitions and assessment tools. However, some expressed sympathy for and solidarity with GPs in this regard, noting that clinical decision making must be difficult given the condition’s limited evidence base.

“*Yeah*, *but I think they need to have more information as well*, *because at this point*, *what has the research shown*? *Like how can you train GPs (to manage long COVID) at this moment in time when there’s not enough knowledge around that*? *I think it’s very difficult for them*.”

- *Patient 4*

In summary, it was apparent that standardised long COVID care solutions are strongly desired by GPs and should be considered as part of long COVID policy initiatives and intervention research going forward.

#### Theme 3—Choosing the right path

While standardising long COVID care pathways was generally viewed favourably, the interviews with GPs and patients offered little in terms of demonstrating which pathways are most effective. Patients were referred to secondary care by GP or emergency services, and usually via one of two pathways, either to hospital specialists or hospital based long COVID clinics. Comparing the quality of patients’ reported care journeys was difficult as experiences varied considerably between patients in terms of the symptoms they had and the healthcare personnel that they encountered.

“…*it’s often a case of just referring maybe to respiratory clinic or cardiology clinic depending on their symptoms*, *and you know it worked out okay*. *They do have to wait awhile for appointments*, *but that’s the same for everybody and they were appropriately assessed*, *and we get the reports back*…”

–*GP 7*

“…*it’s (the Long COVID clinic) not too bad*. *Our patients who have long symptoms who were in-patients were automatically brought back to the long COVID clinics in a nearby hospital and the communication from there is pretty good*…*and patients seem to be happy with how they’re being followed up until they have improved enough to be discharged*.”

- *GP 6*

The accessibility of care options was also constraining in this regard.

“*(I probably spent) …no more than two months overall in the (health) system in terms of getting everything checked out which I thought was pretty good*, *the joys of private health insurance*.”

–*Patient 7*

“…*the only thing is the waiting lists are so long for these things (secondary care appointments)* …*unless you can afford to go private*, *which a lot of people in this day and age can’t*, *they’re too expensive*.”

- *Patient 5*

These findings indicate that further research investigating the merits of long COVID referral options and integrated care pathways may be beneficial.

#### Theme 4—Supportive and collaborative doctor-patient relationships

The interviews showed that the doctor-patient relationship is another major factor underpinning the perceived quality of care that patients receive. Both GPs and patients endorsed the mental health benefits of supportive GP care for patients with long COVID. Valued GP supports that were mentioned included active, sympathetic, and non-judgemental forms of listening.

“*(It’s important for GPs) …to see the patient*, *to speak with them properly*, *to understand what they’re saying*, *to listen to what they’re saying…*”

–*Patient 2*

The offering of practical lifestyle advice and reassurance for patients struggling with the condition’s uncertain course were also endorsed.

*“Try to encourage them to be patient and improve their self-care in terms of diet*, *resting exercise*, *moderate exercise*, *or whatever*, *depending on what they are able for*.”

–*GP 7*

As was being an advocate for patients seeking additional health and social supports.

“*I think (it’s good) if the patient can find a doctor who is sympathetic*, *who’s not critical or dismissive and who is prepared to be inventive and creative in terms of solutions*. *And who will provide the sort of*, *and shall we say administrative*, *that’s not quite the right word*, *but societal or legal supports*.”

–*GP 14*

*Sub-theme i—Empowering patients*. Ensuring that patients’ voices are heard with regard to long COVID service design and planning was often cited by GPs as a positive step forward for long COVID care.

“*I think that the (health) system needs to be designed around the people who are using the service in order to meet their needs*, *and long COVID should be no exception to that*.”

–*GP 9*

“…*my sense is that what works best is for the patient to become expert in these kind of situations…and that we (GPs) accept their subjective experience as the barometer of how they’re doing*, *and that we expect them to help us to help them*, *that they will educate us as to what works for them*, *and what they’ve learned*, *and what other people are doing and that we would*, *you know*, *be very open to assisting them with whatever resources we have*.*”*

–*GP 14*

The more self-efficacious patients in the study further highlighted the considerable merits of empowerment. Their resilience and assertiveness highlighted the positive effects that empowerment can have regarding obtaining access to care services, recovery, and positive mental health.

“*I do think it’s (success at accessing different health services) down to me being so proactive and thanking people as well*, *because as far as I’m concerned*, *doctors are doing a job*, *I’ve got my job*, *they’ve got their job—I like when people thank me for doing my job*, *you know*, *listen*, *thanks very much for that*.”

- *Patient 4*

While mentioned less frequently, the problematic aspects of integrating patient input and clinical decision-making were also discussed by some GPs, particularly around matters like establishing a long COVID diagnosis.

“*You know if someone comes in*, *with a predestined diagnosis*, *that they have long COVID*, *the psychological aspects of thinking they have long COVID*, *has set them into sort of a box in terms of a sick role and all of that*, *and challenging a fixed belief*, *it can take a while for them (patients) to trust you on it*.”

–*GP 12*

This was also an issue with regards to patients that challenged the merits of COVID-19 vaccination.

“*I have also a cohort of people who have come into me and said I haven’t been right since I had my vaccination… there is a phenomenon out there where people believe that they have been damaged by one of their vaccines*. *It’s a small number*, *but they’re there and they’re vocal*. *It’s very hard to dissuade them*.”

–*GP 5*

Patients that sought unnessecary GP referrals to diagnostic imaging services was also problematic in this sense.

“*And this patient had to have every investigation under the sun*, *X-rays*, *CT scans*, *checkups with cardiologists*…*All kinds of referrals and investigations*, *none of which were fruitful*.”

–*GP 5*

In all, supportive care that involves working with patients and working to secure their best interests is desired. However, caution should be exercised with regards to the degree to which patients can influence the clinical decision making process. Collaboration between GPs and patients should be constructive and not negate the value of GP expertise.

## Discussion

### Summary of key findings

This study aimed to answer the questions—“What has been the experience of long COVID in general practice?” and “How can general practice enhance long COVID care and care outcomes?” Using the constant comparison technique, key findings showed that there was strong agreement among interviewed GPs and patients around the complexity of long COVID health problems and the necessity for holistic and integrated multidisciplinary supports. GPs were more vocal than patients about the value of standardising long COVID care operations. Several GPs and patients endorsed the need for supportive and collaborative doctor-patient relationships, although GPs expressed concerns regarding patient influence over some aspects of clinical decision making. There was no agreement among GPs or patients regarding optimal external care supports that GPs can refer patients to.

### Comparisons with existing literature

These findings reinforce many of those from previous primary care long COVID research, particularly findings endorsing the need for holistic and longitudinal care solutions provided by primary care physicians and integrated multidisciplinary healthcare teams [2, 27, 29], mainstreaming of standardised long COVID care solutions to support practice operations [12, 30]; and supportive and collaborative relationships between doctors and patients [20, 33]. Thus, there is certainly sufficient evidence to support interventions prioritising these areas. Meanwhile, this study’s findings contrast with those of much previous research in the area as they provide a close focus on the perspectives of general practice stakeholders. And while the findings of studies conducted by Jamoulle et al. [10], Rotar Pavlic et al. [34], and Taylor et al. [35] also focused on long COVID in general practice, we believe this study is the first to examine both stakeholder groups simultaneously using a comparative analysis technique. By using the constant comparison method, we were able to highlight previously unpublished points of agreement between GPs and patients regarding long COVID care, as well as differences between the priorities that both groups held. This information may prove useful in terms of aligining long COVID interventions with key stakeholders’ priorities, thus enhancing the interventions’ acceptability. The findings also offer what we believe are the first published insights from qualitative research regarding long COVID management in Irish general practice. As such, the findings may be an important source of direction for clinicians, policymakers, and researchers developing long COVID interventions in this country.

### Methodological strengths and limitations

The qualitative approach adopted in this study facilitated close examination of stakeholders’ views on long COVID management in general practice. The Heidegerrian phenomenological theory, RTA, and SRQR frameworks allowed for theory driven and rigorous data analysis, and the constant comparison analytical approach facilitated the capturing of insights regarding similarities and differences between GP and patient stakeholders perspectives on the the study topic. Context was also aided by the pre-interview brief questionnaire findings which helped establish the study sample’s profile and served as useful discussion prompts to expand on in successive qualitative interviews. An inherent limitation of qualitative research is that whilst its findings are ideally suited to capturing participants’ lived experiences, their sample sizes are typically small and therefore cannot be generalised confidently to wider populations. This criticism also applies to this study as the sample was relatively small, particularly the patient sample which did not achieve data saturation, and certainly compared to typical sample sizes in quantitative studies. A more generalisable picture of the research topic will be acquired if this study’s findings are complemented by those of future research with larger samples. Another limitation is the study’s cross-sectional design. Long COVID is characterised by ongoing illness and the course of patients’ symptoms and care experiences can vary considerably between individuals, thus necessitating research involving patient follow-up. Future research using longitudinal designs may address this limitation.

### Implications for research, practice, and policy

This study’s findings demonstrate the need for continued research investigating management of long COVID in general practice. The results suggest that initiatives (e.g. research & education programmes) informing and evaluating GP interventions to enhance consultation processes including long COVID diagnosis, monitoring, and treatment, as well as patient experiences, are especially needed. Studies examining stakeholder perspectives that have larger and /or longitudinal samples may be insightful. Interventions may benefit from a targeted approach focusing on stakeholder groups’ priority concerns. For instance, interested GPs and patients may be encouraged, and where necessary, trained to be involved in studies as researchers as well as participants, thus better ensuring that studies conducted are aligned with their priority concerns. GP targeted interventions may encompass initiatives to enhance standardisation of long COVID consultations (e.g., using standardised definitions and symptom assessment tools), whereas patient level interventions may involve efforts to promote self-efficacy (e.g., self-management support interventions). On a policy level, this study’s findings provide further evidence highlighting the need for focused long COVID care policies. Meanwhile, physician readers may benefit from this study’s findings by incorporating recommended interventions into their routine long COVID care practices.

## Conclusions

This study highlighted the value of structured, integrated, and collaborative care for patients with long COVID in Irish general practice. Clinicians are advised to incorporate these principles into their routine long COVID care practices. Future research with larger and longitudinal samples examining stakeholders’ perspectives are advised to inform the development and evaluation of policy targeted interventions to promote long COVID care in communities.

## Supporting information

S1 Appendix Brief questionnaire (GPs). (DOCX)

S2 Appendix Brief questionnaire (Patients). (DOCX)

S3 Appendix Interview topic guide (GPs). (DOCX)

S4 Appendix Interview topic guide (Patients). (DOCX)
