# Supplementary material for: Enhancing long COVID care in general practice: A qualitative study
Source: PLoS One. 2024 Jun 26;19(6):e0306077. doi: 10.1371/journal.pone.0306077 (PMC11207167; doi:10.1371/journal.pone.0306077)
Supplement: S1 Appendix — (DOCX) [file pone.0306077.s001.docx]

**Appendix A: Brief questionnaire (GPs)**

***1. Age***

___________

***2. Sex***

Male

Female

Other (specify) _________________

***3. General practice location***

__________________________________

***4. Total number of patients at the practice***

________________

***5. Total number of COVID-19 patients at the practice (positive PCR test or clinically diagnosed with COVID-19)***

________________

***6. Total no. of GPs working at the practice***

________________

***7. Which of the following other health professionals work at the practice? (tick all that apply)***

Practice nurse

Psychologist/counsellor

Occupational therapist

Home helper

Physiotherapist

Other (specify) _________________________________

***8. Please say on a scale of 1-5 how much you agree with the following statement, with one being strongly disagree and 5 being strongly agree –***

***“I am satisfied with the COVID-19 related care provided to patients at the practice.”***

Strongly disagree (1)

Disagree (2)

Neither agree nor disagree (3)

Agree (4)

Strongly agree (5)

***9. Would you like to see any of the following initiatives introduced at your practice for Long Covid care? (tick all that apply)***

More patient input / involvement in the care process

Use of Long Covid assessment tools (e.g., questionnaires)

Closer links with specialist care services (e.g., hospital consultants, post-COVID clinics, etc.) for Long Covid patients

More remote care options (e.g., telephone & Skype/Zoom consults)

Therapeutic initiatives (e.g., talk therapy, art therapy, meditation supports, lifestyle recommendations)

Other (please specify)

_____________________________________________________________________
