# Supplementary material for: Enhancing long COVID care in general practice: A qualitative study
Source: PLoS One. 2024 Jun 26;19(6):e0306077. doi: 10.1371/journal.pone.0306077 (PMC11207167; doi:10.1371/journal.pone.0306077)
Supplement: S2 Appendix — (DOCX) [file pone.0306077.s002.docx]

**Appendix B: Brief questionnaire (Patients)**

***1. Age***

___________

***2. Gender***

Male

Female

Other (please specify)

______________________________________________________

***3. Please indicate how much you agree with the following statement, from strongly disagree to strongly agree –***

***“Acute COVID-19 (i.e., the first 2-3 weeks of COVID-19 infection) made me feel unwell.”***

Strongly disagree

Disagree

Neither agree nor disagree

Agree

Strongly agree

***4. Please indicate how much you agree with the following statement, from strongly disagree to strongly agree –***

***“Long COVID made me feel unwell”***

Strongly disagree

Disagree

Neither agree nor disagree

Agree

Strongly agree

***5. Please indicate the approximate length of time that you have experienced Long COVID issues.***

2-4 weeks

4-12 weeks

12 weeks – 6 months

6 months – 1 year

More than 1 year

***6. Please indicate how much you agree with the following statement, from strongly disagree to strongly agree –***

***“Long COVID negatively impacted my physical health”***

Strongly disagree

Disagree

Neither agree nor disagree

Agree

Strongly agree

If relevant, please briefly explain how Long COVID negatively impacted your physical health

______________________________________________________________________________________________________________________________________________________

***7. Please indicate how much you agree with the following statement, from strongly disagree to strongly agree –***

***“Long COVID negatively impacted my mental health”***

Strongly disagree

Disagree

Neither agree nor disagree

Agree

Strongly agree

If relevant, please briefly explain how Long COVID negatively impacted your mental health

______________________________________________________________________________________________________________________________________________________

***8. Please indicate how much you agree with the following statement, from strongly disagree to strongly agree –***

***“Long COVID negatively impacted my quality of life”***

Strongly disagree

Disagree

Neither agree nor disagree

Agree

Strongly agree

If relevant, please briefly explain how Long COVID negatively impacted your quality of life

______________________________________________________________________________________________________________________________________________________

***9. Have you spoken to a General Practitioner (GP) about Long COVID problems that you have had?***

No

Yes

***10. Please indicate how much you agree with the following statement, from strongly disagree to strongly agree –***

***“I am satisfied with the Long COVID care I have received from my GP.”***

Strongly disagree

Disagree

Neither agree nor disagree

Agree

Strongly agree

***11. Which of the following would you like to see introduced by your GP for care of long- Long COVID problems (tick all that apply)***

More patient input / involvement

Use of Long COVID screening/diagnosis tools (e.g., questionnaires)

Better links with specialist services (e.g., hospital services)

More remote care options (e.g., telephone, Skype, Zoom)

Mental health support (e.g., talk therapy, lifestyle recommendations etc.)

Other (please explain below)

_____________________________________________________________________ _____________________________________________________________________
