# Supplementary material for: Enhancing long COVID care in general practice: A qualitative study
Source: PLoS One. 2024 Jun 26;19(6):e0306077. doi: 10.1371/journal.pone.0306077 (PMC11207167; doi:10.1371/journal.pone.0306077)
Supplement: S3 Appendix — (DOCX) [file pone.0306077.s003.docx]

**Appendix C : Interview topic guide (GPs)**

1. What has your experience of Long Covid in general practice been like?
2. Have Long Covid issues been common amongst your patients?
3. What kind of physical health / mental health issues have your Long Covid patients had?
4. What kind of care have you provided for your Long Covid patients to address these issues?
5. Is there anything you would change about your (and general practice’s more broadly) current approach to Long Covid care?
6. Do you think there is a need for new initiatives in general practice to combat Long Covid in communities?
7. What kind of initiatives do you think would be effective and why?
