# Supplementary material for: Enhancing long COVID care in general practice: A qualitative study
Source: PLoS One. 2024 Jun 26;19(6):e0306077. doi: 10.1371/journal.pone.0306077 (PMC11207167; doi:10.1371/journal.pone.0306077)
Supplement: S4 Appendix — (DOCX) [file pone.0306077.s004.docx]

**Appendix D : Interview topic guide (Patients)**

1. Did you experience what is known as Long Covid?
2. How was your experience of Long Covid?
3. Did you seek care (especially GP care) for Long Covid health / mental health issues?
4. If yes, how was your experience of the care provided?
5. Is there anything you would change about the Long Covid care you received?
6. Did the care you received help you overcome problems linked to Long Covid?
7. Do you think there is a need for initiatives in general practice to combat Long Covid in communities?
